# Efficiency of private and public primary health facilities accredited by the National Health Insurance Authority in Ghana

**DOI:** 10.1186/s12962-015-0050-z

**Published:** 2015-12-26

**Authors:** Robert Kaba Alhassan, Edward Nketiah-Amponsah, James Akazili, Nicole Spieker, Daniel Kojo Arhinful, Tobias F Rinke de Wit

**Affiliations:** Department of Epidemiology, Noguchi Memorial Institute for Medical Research (NMIMR), University of Ghana, Legon, P.O Box LG 583, Accra, Ghana; Amsterdam Institute for Global Health and Development, University of Amsterdam, Amsterdam, The Netherlands; Department of Economics, University of Ghana, Legon, Ghana; PharmAccess Foundation, Amsterdam, The Netherlands; Health Research Unit of Ghana Health Service, Navrongo Health Research Centre, Navrongo, Ghana

**Keywords:** Efficiency, Quality care, Primary health facilities, Health insurance, Sustainability, Ghana

## Abstract

**Background:**

Despite improvements in a number of health outcome indicators partly due to the National Health Insurance Scheme (NHIS), Ghana is unlikely to attain all its health-related millennium development goals before the end of 2015. Inefficient use of available limited resources has been cited as a contributory factor for this predicament. This study sought to explore efficiency levels of NHIS-accredited private and public health facilities; ascertain factors that account for differences in efficiency and determine the association between quality care and efficiency levels.

**Methods:**

The study is a cross-sectional survey of NHIS-accredited primary health facilities (n = 64) in two regions in southern Ghana. Data Envelopment Analysis was used to estimate technical efficiency of sampled health facilities while Tobit regression was employed to predict factors associated with efficiency levels. Spearman correlation test was performed to determine the association between quality care and efficiency.

**Results:**

Overall, 20 out of the 64 health facilities (31 %) were optimally efficient relative to their peers. Out of the 20 efficient facilities, 10 (50 %) were Public/government owned facilities; 8 (40 %) were Private-for-profit facilities and 2 (10 %) were Private-not-for-profit/Mission facilities. Mission (Coef. = 52.1; p = 0.000) and Public (Coef. = 42.9; p = 0.002) facilities located in the Western region (predominantly rural) had higher odds of attaining the 100 % technical efficiency benchmark than those located in the Greater Accra region (largely urban). No significant association was found between technical efficiency scores of health facilities and many technical quality care proxies, except in overall quality score per the NHIS accreditation data (Coef. = −0.3158; p < 0.05) and *SafeCare Essentials* quality score on environmental safety for staff and patients (Coef. = −0.2764; p < 0.05) where the association was negative.

**Conclusions:**

The findings suggest some level of wastage of health resources in many healthcare facilities, especially those located in urban areas. The Ministry of Health and relevant stakeholders should undertake more effective need analysis to inform resource allocation, distribution and capacity building to promote efficient utilization of limited resources without compromising quality care standards.

## Background

Within the West African sub-region, Ghana is performing relatively better than its neighbors on most health indicators. As at 2012, life expectancy at birth was 61 years compared to 56 in Burkina Faso; 50 in Cote d’Ivoire and 56 in Togo [1]. Likewise, under-five mortality was 69 per 1000 live births in Ghana compared to 166 in Burkina Faso and 119 in Cote d’Ivoire [2].

Notwithstanding these achievements, limited health resources keep confronting the country in meeting its health targets including the health-related millennium development goals (MDGs) [3], now termed sustainable development goals (SDGs). This necessitates more efficiency at all levels of the health system especially at the primary healthcare level where resources are more scarce, albeit over 50 % of the Ghanaian population access basic healthcare at this level.

Ghana’s healthcare system is divided into three administrative levels namely national, regional and district. At the national level, the Ministry of Health (MoH) is responsible for policy formulation and resource mobilization while the national headquarters of the Ghana Health Service (GHS) is responsible for policy implementation through the regional and district health administrations. At the regional level, the regional health administration (RHA) is responsible for administration of health services delivery in a particular region and supervises activities of the district health management teams (DHMTs). The district level is managed by the DHMTs. District hospitals, health centres, clinics and community-based health planning and services (CHPS) compounds are monitored and supervised by the DHMTs.

In Ghana, formal health service delivery is executed by 4 teaching hospitals, 9 regional hospitals, 3 psychiatric hospitals, 343 district hospitals, over 2000 clinics, health centres and polyclinics. In terms of ownership, there are 1607 government owned facilities, 91 quasi-government, 245 mission and 1277 private-for-private facilities [4].

Primary healthcare services are rendered by primary providers such as health centres, clinics, CHPS compounds and traditional healers. Health centres and clinics usually serve a community with a population of 15,000–30,000 people with basic curative care, disease prevention, maternal and child health services. Referral cases from the primary healthcare level are sent to district and regional hospitals or teaching hospitals where specialized clinical and diagnostic care are rendered.

Barely a decade after introduction of the National Health Insurance Scheme (NHIS), nearly 40 % of the Ghanaian population are in active membership thus remedying the spiraling cost of healthcare for households and individuals [5, 6]. From 2009 to 2013, a cumulative number of 3828 health facilities have been given full accreditation by the National Health Insurance Authority (NHIA) (regulatory body of the NHIS); out of this number, 1203 (31 %) were clinics and health centres; approximately 54 % were government owned, 6 % were mission; nearly 40 % were private-for-profit; 1 % were quasi-government institutions [5].

Even though the NHIS has contributed significantly to improved out-patient and in-patient attendance, reduced maternal mortality rates and increased percentage of skilled deliveries in the country [7], there are increasing concerns with respect to its operational and financial sustainability [5, 8–13]. Besides the escalating cost of claims payment [5], operational inefficiencies in private and public health facilities are cited as potential sustainability threats to the NHIS [14].

Attainment of acceptable efficiency levels in private and public health facilities is captured as a core objective in a number of Ghanaian national policy documents and reports [7, 14–17] albeit limited scientific knowledge exists on technical efficiency and facility ownership, especially in the context of NHIS-accredited facilities.

Though some empirical studies have been conducted on technical efficiency of health facilities in Ghana [18–20] and other countries in Africa [21–25], these studies did not explore differences in private and public facilities. Publication by Jehu-Appiah et al. [4] is one known publication on Ghana that compared technical efficiency levels in private and public health facilities. Nonetheless, Jehu-Appiah et al. [4] analyzed 2005 data which might not reflect the current situation in sampled facilities. Moreover, health facilities used by Jehu-Appiah et al. [4] were not accredited since formal NHIS accreditation was initiated in 2009.

Given the limited empirical studies on facility ownership and technical efficiency in Ghana, this paper sought to quantify the technical efficiency levels of 64 NHIS-accredited private and public primary health facilities in Ghana and determine what factors account for possible differences. The association between efficiency and quality care is also explored. The hypothesis is that facility ownership has a significant association with efficiency levels, holding other facility characteristics constant.

Findings of this study are expected to inform policy discussions on possible avenues for leveraging public–private partnership in healthcare delivery to improve efficiency levels without compromising good quality care in Ghana and other sub-Saharan African countries.

## Methods

### Research setting

The study was conducted in Greater Accra (GAR) and Western (WR) regions of southern Ghana. The GAR is predominantly urban and cosmopolitan with close to 4 million people and 416 NHIS-accredited healthcare facilities. The WR is largely rural with a population of a little over 2 million people and 438 NHIS-accredited health facilities [15].

### Study design and data collection

This is a cross-sectional study which is part of a four year randomized control trial (RCT) project that assesses client centeredness of Ghana’s healthcare provision and administrative services [26]. Data was collected using a tool called Situational Analysis plus (SA^+^), a component of the SafeCare quality assessment tool kit [27]. The SA^+^ tool collects data on health facility services, activities and assets (human and material). The tool was administered by three (3) trained research assistants, who assessed one health facility at a time.

Besides the SA^+^ tool, quality care delivery in the sampled health facilities was determined using *SafeCare Essentials* tool (see data analysis section) and secondary data on NHIA accreditation scores. Piloting of SA^+^ was done in one conveniently sampled health facility in the GAR to check consistency and accuracy. Data collection lasted from March to June, 2012 in both regions.

Private facilities were operationally defined in this study to include private-for-profit, mission and non-governmental organization (NGO) facilities. Public facilities included government and quasi-government facilities. Primary health facilities are referred to clinics and health centres that mainly render “first-point-of-call” outpatient services.

An NHIS-accredited health facility is a facility that has been assessed by the NHIA based on 11 predetermined standard areas and several sub-assessment criteria/questions. Once a facility meets the required standards, an accreditation certificate is issued for five (5) years on first instance and subsequently renewed every two (2) years. In Ghana, only NHIS-accredited facilities are allowed by law to render services to NHIS subscribers and later submit claims to the NHIA for reimbursement [15].

### Sampling procedures

Multi-stage sampling technique was adopted where GAR and WR were purposively sampled for rural–urban balance. This was followed by purposive sampling of eight (8) districts from each region after a Principal Component Analysis (PCA). Variables used for the PCA were: average NHIS accredited grade of health facilities in a district; NHIS enrolment rate; district population, and number of accredited and non-accredited health facilities. Likewise, PCA scores were generated for all accredited primary health facilities in the two regions and 32 facilities with closest scores were sampled from each region, making a total of 64. This ensured homogeneity in the sampled health facilities, which is needed to detect effect of implemented interventions by the RCT. Next, each district was proportionally allocated maximum of 4 facilities. The 32 facilities in GAR and WR represented approximately 8 % of the total number of accredited facilities in each region during the survey. Hospitals and other higher level health facilities were exempted from the study because they are relatively complex for impact evaluation.

### Ethical considerations

Ethical clearance for the survey was obtained from the Ghana Health Service (GHS) Ethical Review Committee (ERC) (clearance number: GHS-ERC: 18/5/11). Moreover, written informed consent was sought from health facility heads, the district and regional health directorates.

### Measuring efficiency using the DEA approach

In the literature two principal approaches are used to measure efficiency of firms (including health facilities) namely: Data Envelopment Analysis (DEA) and stochastic frontiers [28]. The DEA model first proposed by Charnes et al. [29] involves the use of linear programming methods to construct non-parametric frontier over the data, while the stochastic frontier is an econometric approach [30].

The DEA approach is used to benchmark performance and the relative efficiency of each production unit among a set of fairly homogeneous Decision Making Units (DMUs), such as clinics and health centres that use similar inputs to produce service outputs. DMUs deemed optimally efficient among their peers (based on available inputs and outputs) are assigned an efficiency score of 1.0 which is equivalent to 100 % in percentage terms.

A health facility is described as fully efficient among its peers when it attains an efficiency score of 1.0 and completely inefficient when it attains an efficiency score of 0.0 (equivalent to 0 % in percentage terms). It must be emphasized that facilities estimated as optimally efficient among peers might not necessarily be efficient in absolute terms. There is therefore the need to interpret results of the DEA in the context of relative efficiency of facilities under assessment.

Measurement of efficiency can be technical or allocative and the orientation can be input-orientated or output-orientated; details of these distinctions can be found in Coelli [28]. For the purposes of this study, the focus was on technical efficiency because there was adequate data on the input and output factors of interest. Allocative efficiency was not considered because of inadequate data on cost of services and revenue of the selected 64 facilities, a challenge acknowledged by Akazili et al. [19] and Kirigia et al. [22] in their studies on Ghana and Benin respectively.

Technical efficiency (TE) of a DMU is defined as TE = *Weighted sum of outputs divided by weighted sum of inputs* [30]. The current analysis used input-orientated technical efficiency measures assuming constant returns to scale (CRS) and variable returns to scale (VRS) [29]. This approach was used because in Ghana health centres and clinics have some level of control over inputs than outputs [19]. The VRS approach helped determine whether a DMU’s production exhibits increasing returns to scale, constant returns to scale or decreasing returns to scale.

The DEA is a nonparametric statistical test that has been used as a standard method to estimate technical efficiency within and outside Ghana because of some advantages over the stochastic frontiers [4, 19, 20]. The technical efficiency score of each clinic and health centre was attained by solving models 1 and 2 below as presented in a similar study by Osei et al. [20].

**Table:** 

DEA weights model 1: input-orientated, CRS	DEA weights model 2: input-orientated, VRS
${\text{Eff}} = {\text{Max}} \mathop \sum \limits_{\text{r}} {\text{u}}_{\text{r}} {\text{y}}_{{{\text{rj}}0}}$ ${\text{u}}_{{{\text{r}} '}} {\text{v}}_{\text{i}}$ *s.t.* $\mathop \sum \limits_{\text{r}} {\text{u}}_{\text{r}} {\text{y}}_{\text{rj}} - \mathop \sum \limits_{\text{i}} {\text{v}}_{\text{i}} {\text{x}}_{{{\text{ij }} \le 0 ; }} \forall {\text{j}}$ $\mathop \sum \limits_{\text{i}} {\text{v}}_{\text{i}} {\text{x}}_{{{\text{ij}}0 }} = 1$ ${\text{u}}_{\text{r '}} {\text{v}}_{\text{i}}$ ≥ 0 $;$ $\forall {\text{r}}, \forall {\text{i}}.$	${\text{Eff}} = {\text{Max }}\mathop \sum \limits_{\text{r}} {\text{u}}_{\text{r}} {\text{y}}_{{{\text{rj}}0 + {\text{u}}_{0} }}$ ${\text{u}}_{\text{r, }} {\text{v}}_{\text{i}}$ *s.t.* $\mathop \sum \limits_{\text{r}} {\text{u}}_{\text{r}} {\text{y}}_{\text{rj}} - \mathop \sum \limits_{\text{i}} {\text{v}}_{\text{i}} {\text{x}}_{{{\text{ij }} + {\text{u}}_{0} \le 0 ; }} \forall {\text{j}}$ $\mathop \sum \limits_{\text{i}} {\text{v}}_{\text{i}} {\text{x}}_{{{\text{ij}}0 }} = 1$ ${\text{u}}_{\text{r '}} {\text{v}}_{\text{i}}$ ≥ 0 $;$ $\forall {\text{r}}, \forall {\text{i}}.$

where: *Y*_*rj*_ = the amount of output *r* produced by clinic or health centre *j*, *X*_*ij*_ = the amount of inputs *i* used by clinic or health centre *j*, *u*_*r*_ = the weight given to output *r* (*r* = 1,……, *t* and *t* is the number of outputs), *v*_*i*_ = the weight given to input *i*, (*i* = 1,……., *m* and *m* is the number of inputs), *n* = the number of clinics or health centres, *j*_*0*_ = the number of clinics or health centres under assessment.

### Advantages of the DEA

The DEA approach to frontier estimation has been argued to accommodate multiple inputs and outputs in a single measure of efficiency unlike the Stochastic Frontier Analysis (SFA) which cannot [29, 30]. Unlike the parametric frontier models, the DEA does not suffer from the problem of model mis-specification which could possibly present misleading results, Charnes et al. [30]. Furthermore, Akazili et al. [19] argued that the DEA does not suffer from problems of multicollinearity and heteroscedasity as seen in SFA.

### Limitations of the DEA

The DEA model potentially justifies inefficiency in DMUs since a DMU can be efficient among its peers but actually be inefficient in absolute terms [19, 28]. Secondly, since the DEA is not a parametric statistical method, hypothesis testing could be a challenge [19]. The DEA is primarily a diagnostic tool and does not necessarily prescribe strategies to make inefficient firms efficient. These limitations can however, be controlled with large sample size representative of the population and use of complementary analysis such as Tobit regression [31].

### Data analysis and rationale for selecting inputs and outputs

Data analysis was done at two levels. The first level used the Data Envelopment Analysis Programme (DEAP) version 2.1 to estimate the technical efficiency scores of the 64 facilities based on five (5) inputs and four (4) outputs. These input and output factors were considered because of their relevance to primary healthcare which is the main focus and preoccupation of sampled clinics and health centres. These factors were also selected because of their relevance in attainment of the health-related MDGs in Ghana. Moreover, there was adequate data on these input and output factors in the sampled health facilities. The number of inputs (n = 5) and outputs (n = 4) used for the DEA is also consistent with approaches by previous related studies [4, 18–20] to avoid extreme trade-off between estimated efficiency and number of inputs and outputs used.

Another criteria used for the inputs selection was their relevance to clinic/health centre settings. Because clinics/health centres are smaller in size and scope, observation beds, wards (mainly for observing basic medical conditions for less than 24 h and referred if complicated), consulting rooms, clinical and support staff were considered for the DEA; the selection criteria would have been different if the facilities were secondary or tertiary hospitals. Since clinics and health centres at the primary healthcare level do not render inpatient services and other complex healthcare services, only relevant output factors such as number of spontaneous vaginal deliveries (SVDs), outpatient attendance, number of antenatal, postnatal, and reproductive services were considered. The input and output factors were thus carefully selected to reflect the capacity and scope of sampled facilities in clinical and non-clinical activities. Below are the input and output factors:

**Table:** 

Input factors	Output factors (per month)
1: Number of clinical staff	1: Number of deliveries
2: Number of support staff	2: Number of out-patient visits
3: Number of observation beds	3: Number of antenatal and postnatal visits
4: Number of detention wards	4: Number of family planning (FP), reproductive and child health (RCH) visits
5: Number of consulting rooms	

The second level of analysis was a Tobit regression and Spearman rank correlation analysis using STATA (version 12.0) to predict factors associated with efficiency levels in health facilities. Advantages of using Tobit regression over other approaches have been indicated in Long [31]. For instance, when variables are censored, Tobit regression is able to provide better consistent estimates of the parameters than the truncated regression. The dependent variable of interest for the Tobit regression was technical efficiency score of health facilities which was right-censored using the upper limit of 1.0 (equivalent to 100 %), thus facilities that fall below this limit were deemed inefficient relative to their peers.

The independent variable of interest was facility ownership which was categorized into “private-for-profit”, “public/government” and “mission/NGO”. Control variables in the regression model were health facility rural–urban location, gender of health facility manager/owner, and presence of complaint system. These control variables were intuitively selected because of their potential effect on administrative effectiveness or otherwise which in turn could influence facility efficiency levels. The Tobit regression was modeled as follows using maximum likelihood, assuming homoskedastic normal disturbances:

$$Tobit\left( {yj} \right) = \alpha 0 + \alpha 1xj1 + \alpha 2xj2 + \alpha 3xj3 + \cdots \cdots + \varepsilon j$$

where: *yj* is the constant returns to scale efficiency score for the *jth* health facility, *xj* are the independent variables, $\alpha$ is the coefficient and *ɛj* are the disturbance term assumed to be normally distributed with the μ mean and standard deviation σ. Multicollinearity diagnostics was performed for all explanatory variables prior to their inclusion in the regression model and none had a variance inflation factor (VIF) up to the 10.0 rule of thumb necessary for exclusion [32].

Spearman rank correlation test was performed to ascertain the association between technical efficiency scores and quality care proxies using the NHIA five core standard areas and the *SafeCare Essentials* patient risk areas. The pair-wise correlation coefficients were determined at 95 % confidence level.

For the purposes of this paper, quality care was determined using the NHIA accreditation data on performance of the 64 sampled health facilities on five core standard areas namely: range of services, staffing, organization and management, safety and quality management, and service delivery. Besides the NHIA core standard areas, the authors used an assessment tool kit called *SafeCare Essentials* [27]. The *SafeCare Essentials* tool is provided by the SafeCare Initiative, a collaboration of the PharmAccess Foundation in The Netherlands, the Council for Health Services Accreditation of Southern Africa (COHSASA), and the Joint Commission International (JCI), United States (US). The tool aims at identifying the capability of a facility to move slowly or more rapidly towards higher levels of clinical quality and safer patient care according to staff efforts [27].

The *SafeCare Essentials* tool comprises of 41 assessment criteria categorized into five risk areas, namely: leadership and accountability (7 criteria); competent and capable workforce (7 criteria); safe environment for staff and patients (10 criteria); clinical care of patients (10 criteria), and improvement of quality and safety (7 criteria). Each assessment criteria is scored on a four-point scale (0–3) called levels of effort. High levels depict better efforts by staff of pertinent health facility towards enhancing patient safety and reducing risk (see Alhassan et al. [26]).

## Results

### Characteristics of health facilities

All 64 clinics and health centres fully participated in the study representing a return rate of 100 %. As shown in Table 1, nearly 50 % of the health facilities were private-for-profit; 41 % were public/government owned and 12 % were mission/NGO facilities. Close to 60 % of the facilities were located in rural areas; 55 % were either owned or managed by males; 55 % did not receive any form of donor funding support; 78 % had no functional computers in place and 92 % had no active complaint systems for clients.

**Table 1:** Characteristics of surveyed clinics and health centres (n = 64)

Facility characteristics	Descriptive statistics	
	Frequency (f)	Percentage (%)
Ownership		
Private-for-profit	30	47
Public/government	26	41
Mission/NGO	8	12
Geographical location		
Rural	36	56
Urban	28	44
Region		
Greater Accra	32	50
Western	32	50
Gender of facility owner/manager^a^		
Male	35	55
Female	29	45
Facility receives donor funds		
Yes	29	45
No	35	55
Facility has functional computer(s)		
Yes	14	22
No	50	78
Presence of active complaint system for clients		
Yes	5	8
No	59	92

WOTRO-COHEiSION Ghana Project (Health Facility Survey Data: March–June, 2012)

^a^Facility “owner” applies in the case of private facilities; “manager” applies to both public and private facilities. Some private facility managers are also the owners; public facilities are always owned by the Ghana Health Service or Ministry of Health or quasi-government body

On the average, there were more clinical staff (mean = 16, SD = 14) than support staff (mean = 8, SD = 9) per health facility. The average number of observation beds in a health facility was 11 (SD = 10) while the average number of wards and consulting rooms per health facility was 2. The dominant service rendered was outpatient visits (mean = 1011, SD = 787) followed by antenatal (ANC)/postnatal (PNC) visits (mean = 512, SD = 712) and family planning (FP)/reproductive and child health (RCH) visits (mean = 208, SD = 355); the number of SVDs per month per health facility was 13 (SD = 16) (see Table 2). Public and private facilities did not significantly differ in their inputs and outputs records, apart from private facilities having one more ward and public facilities doing more SVDs (19, versus 9), recording more ANC/PNC visits (798, versus 317) and FP/RCH visits (429 versus 56) (see Table 2).

**Table 2:** Human and material resources in health facilities

Input and output variables	Efficiency score			Facility ownership^a^			Total
	1.0 (n = 20)	<1.0 (n = 44)	*p* value	Private (n = 38)	Public (n = 26)	p-value	Mean (SD)
	Mean (SD)	Mean (SD)		Mean (SD)	Mean (SD)		
Input variables							
Number of clinical staff	11(7)	19 (16)	0.0509	14 (13)	19 (15)	0.2256	16 (14)
Number of support staff	4 (4)	9 (11)	0.0426*	8 (11)	7 (6)	0.7297	8 (9)
Number of beds	9 (8)	11 (11)	0.4011	12 (11)	8 (8)	0.1327	11 (10)
Number of wards	2 (1)	2 (1)	0.3320	2 (1)	1 (1)	0.0332*	2 (1)
Number of consulting rooms	1 (0.3)	2 (1)	0.0177*	2 (1)	1 (1)	0.4312	1 (1)
Output variables (per month)							
Number of deliveries	17 (20)	11 (15)	0.1819	9 (15)	19 (17)	0.0115*	13 (16)
Number of OPD visits	1197 (732)	927 (805)	0.2054	1047 (868)	958 (665)	0.6583	1011 (787)
Number of ANC/PNC visits	677 (956)	437 (567)	0.2138	317 (657)	798 (705)	0.0069*	512
Number of FP and RCH visits	321 (480)	156 (273)	0.0841	56 (129)	429 (456)	0.0000*	208 (355)

WOTRO-COHEiSION Ghana Project (Health Facility Survey Data: March–June, 2012)

*FP* family planning, *RCH* reproductive and child health, *OPD* Out-patient department, ANC antenatal care; PNC postnatal care

* Two tail test of hypothesis statistically significant at 95 % confidence level using the Student t test

^a^Ownership is dichotomized for the t test where government and quasi-government facilities are classified under “public” and private-for-profit and Mission/NGO health facilities classified under “private”

### The DEA results

**Fig. 1:**
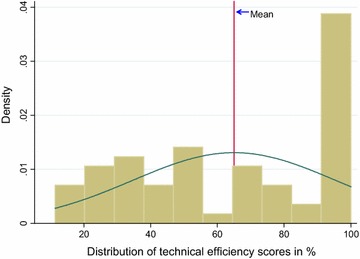
Distribution of technical efficiency scores (n = 64).* Source* WOTRO-COHEiSION Ghana Project (Health Facility Survey Data: March–June, 2012). The figure shows distribution of technical efficiency scores indicating the mean efficiency score with the *red line* and the distribution curve with the *green line*

**Fig. 2:**
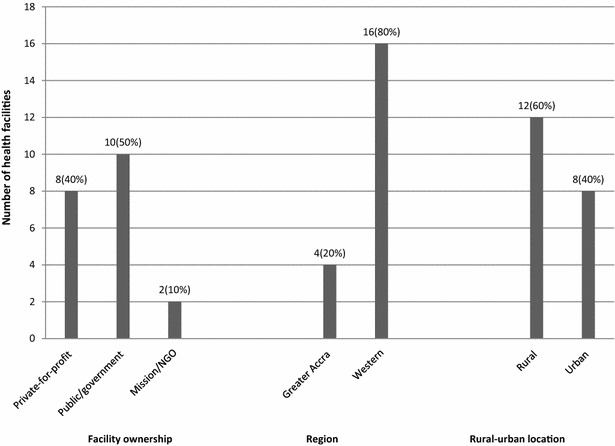
Categories of health facilities operating at optimal efficiency level (n = 20).* Source* WOTRO-COHEiSION Ghana Project (Health Facility Survey Data: March–June, 2012). NGO (Non-governmental organization)

The DEA results showed that the average technical efficiency score for the 64 facilities was 0.65 (i.e. 65 % in percentage terms); 20 facilities (31 %) attained the 1.0 optimal efficiency, and the remaining 44(69 %) attained efficiency scores below 1.0. The lowest efficiency score was 0.11 attained by one health facility (see Fig. 1). Out of the 20 facilities that attained 1.0 efficiency, 2 (10 %) were mission/NGO; 8 (40 %) were private-for-profit; 10 (50 %) were public/government. In terms of the regions, 4 out of the 20 efficient facilities were located in GAR while 16 (80 %) were in WR; 60 % of the efficient facilities were rural-based and the remaining 40 % were urban- based (see Fig. 2).

**Fig. 3:**
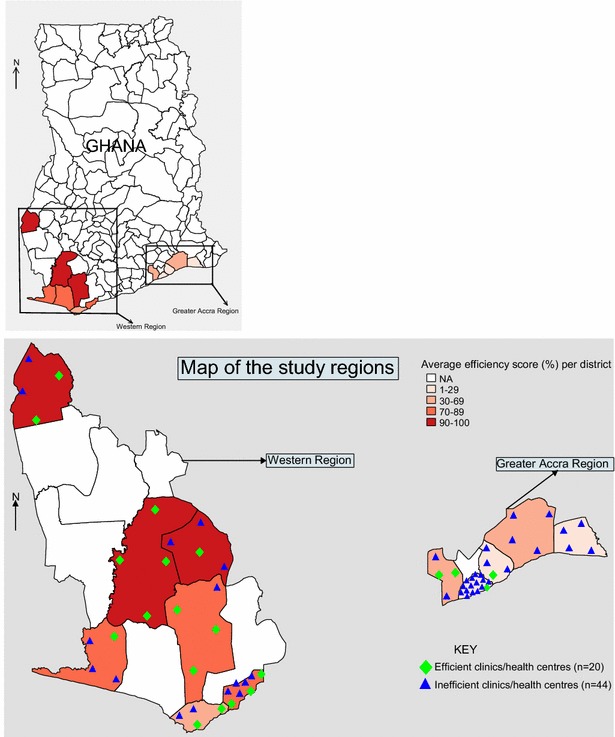
Geographic distribution of efficient and inefficient health facilities.* Source* WOTRO-COHEiSION Ghana Project (Health Facility Survey Data: March–June, 2012)

On the whole, health facilities that attained efficiency scores below the 1.0 efficiency benchmark used excess (surplus) human and material resources but recorded lesser health service activities such as number of SVDs, and outpatient attendance per month. Conversely, health facilities that operated at 1.0 efficiency level had lesser number of clinical staff, support staff, beds and wards though recorded more SVDs, ANC/PNC visits and FP/RCH visits than their inefficient counterparts (p < 0.05) (see Table 2). The results also showed that lower efficiency scores were recorded by many urban health facilities while many rural facilities recorded higher efficiency scores (p < 0.0001) (see Fig. 3). The beds and wards were mainly for observation and primary healthcare services since clinics and health centres (especially public/government owned) do not typically render complex inpatient services. All output factors were thus recorded based on mainstream primary healthcare services.

Table 3 depicts the constant returns to scale (CRS) and the variable returns to scale (VRS) values according to facility ownership. The CRS values are the average efficiency scores while the VRS figures depict the average input excesses that need to be reduced or output targets required to enhance efficiency levels in inefficient facilities. The mean VRS irrespective of facility ownership was 84 %, indicating that 16 % of input reduction or 84 % output increase is needed for attaining the 1.0 optimal efficiency benchmark. Based on facility ownership, inefficient private-for-profit and mission/NGO facilities will need up to 13 % input reduction or 87 % output increases to enhance their efficiency level, given their available material and human resources. Inefficient public/government facilities will need inputs cuts up to 19 % or outputs increases of about 81 % to make them efficient considering their available health resources (see Table 3).

**Table 3:** Average inputs reductions or outputs increases based on facility ownership

Facility ownership	Constant returns to scale (CRS)				Variable returns to scale (VRS)			
	Mean (%)	Std. Dev (%)	Min	Max	Mean (%)	Std. Dev (%)	Min	Min
Private-for-profit (n = 30)	58	33	11	100	87	21	32	100
Public/government (n = 26)	71	30	15	100	81	21	27	100
Mission/NGO (n = 8)	73	20	47	100	87	14	71	100
Total (n = 64)	65	30	11	100	84	20	27 %	100

WOTRO-COHEiSION Ghana Project (Health Facility Survey Data: March-June, 2012)

Constant Returns to Scale (CRS): depict the average efficiency scores by the health facilities based on ownership; Variable Returns to Scale (VRS): depict the average input excesses that need to be reduced or output targets to make inefficient facilities efficient

**Fig. 4:**
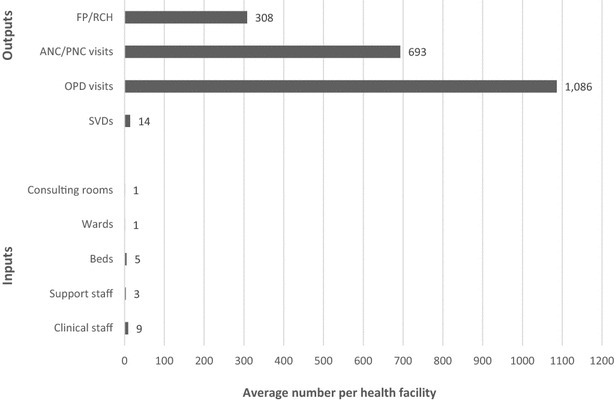
Inputs cuts and outputs increases needed to make facilities attain optimal efficiency (n = 44).* Source* WOTRO-COHEiSION Ghana Project (Health Facility Survey Data: March–June, 2012). OPD (outpatient department); ANC (antenatal care); PNC (postnatal care); FP (family planning); RCH (reproductive and child health); SVDs (spontaneous vaginal deliveries); *Note* facilities shown here are those that attained efficiency scores below 1.0 (or 100 %)

Furthermore, the results showed that each of the health facilities that did not attain optimal efficiency levels will need an average reduction of 9 clinical staff; 3 non-clinical staff; 5 beds, 1 consulting room and, 1 ward from their current assets endowment to make them more efficient. Alternatively, these individual facilities in maintaining their current assets will approximately need to increase their output in SVDs to 14, outpatient visits to 1086, ANC/PNC visits to 693, and FP/RCH visits to 308 to make them more efficient (see Fig. 4).

### Factors associated with health facility efficiency

As shown in Table 4, facility ownership is significantly associated with technical efficiency levels in health facilities. Higher efficiency scores were particularly associated with public/government facilities and Mission/NGO facilities located in WR than those in GAR (p < 0.05). Control variables such as gender of facility owner/manager, presence of complaint systems, access to donor funding, and rural–urban location did not have significant relationship with efficiency levels.

**Table 4:** Factors associated with technical efficiency levels in health facilities (n = 64)

Independent variables	Dependent variable: technical efficiency score^a^		
	Coef.	p value	[95 % Conf. Int.]
Public/government facilities (WR)	42.9	0.002*	16.9 to 69.0
Public/government facilities (GAR)	Ref	Ref	Ref
Mission/NGO facilities (WR)	52.1	0.000*	24.6 to 79.6
Mission/NGO facilities (GAR)	Ref	Ref	Ref
Private-for-profit facilities (WR)	26.3	0.085	−3.8 to 56.4
Private-for-profit facilities (GAR)	Ref	Ref	Ref
Rural facilities	3.87	0.680	−14.9 to 22.6
Urban facilities	Ref	Ref	Ref
Facilities owned/managed by male	−2.03	0.846	−22.8 to 18.8
Facilities owned/managed by female	Ref	Ref	Ref
Facilities with access to donor funding	6.79	0.589	−18.3 to 31.8
Facilities without access to donor funding	Ref	Ref	Ref
Facilities with active client complaint system	1.12	0.953	−36.4 to 38.6
Facilities without active client complaint system	Ref	Ref	Ref
Log Likelihood = −232.58405			
Prob > Chi_2_ = 0.0006			
Pseudo R_2_ = 0.0524			

WOTRO-COHEiSION Ghana Project (Health Facility Survey Data: March–June, 2012)

Greater Accra Region (GAR); WR (Western Region)

* Statistically significant at 0.05 level of significance

^a^Dependent variable (technical efficiency  % score) right-censored at 1.0 (equivalent to 100 %), benchmark for technically efficient facilities. Facilities scoring below 1.0 are considered inefficient

Finally, the results showed that many of the quality care proxies had no significant association with technical efficiency (p > 0.05); thus, high efficiency levels did not necessarily associate with better quality care standards in sampled health facilities and vice versa. Technical efficiency was only significantly associated with one *SafeCare Essentials* risk area (Coef. = −0.2764, p < 0.05) and the overall NHIA quality assessment score (Coef. = −0.3158, p < 0.05) (see Table 5).

**Table 5:** Association between quality care proxies and technical efficiency (n = 64)

Quality care proxies	Technical efficiency score
	Coef.
NHIA core standard areas	
Range of services	−0.1416
Staffing	−0.0522
Organization and management	−0.1370
Quality and safety management	−0.1431
Care delivery	−0.1946
Overall score	−0.3158*
*SafeCare Essentials* patient risk areas	
Leadership and accountability	−0.0834
Competency of workforce	0.0055
Environmental safety	−0.2764*
Clinical care	−0.1318
Quality improvement	−0.0421
Overall score	−0.1912

WOTRO-COHEiSION Ghana Project (Health Facility Survey Data: March–June, 2012)

Quality care proxies represent the health facilities performance in adherence to patient safety and standard quality health service delivery protocols. Higher scores depict better efforts and adherence to these standard protocols and vice versa

* Spearman rank correlation statistically significant at 0.05 level of significance (unadjusted Bonferroni or Sidak)

## Discussion

This study found that approximately 31 % of the 64 sampled facilities were operating more efficiently relative to their peers. Even though several factors might account for this outcome, the efficiency scores distribution could have been skewed by the dominance of rural facilities (n = 36) in the study sample. Since majority of the rural clinics and health centres are less endowed with material and human resources but record huge clinic attendance (see Table 2), it is expected that a preponderance of them will be deemed efficient because the DEA estimations are based on weighted sum of service output divided by weighted sum of inputs (resources). Thus, the higher the outputs over inputs the higher the efficiency score and vice versa. Majority (80 %) of the efficient facilities were in the Western region which is largely rural while the remaining 20 % were in the Greater Accra.

Furthermore, the distribution of efficiency scores (see Fig. 1) could be attributed to the purposive sampling of primary healthcare facilities for this study. Primary facilities, being the frontier of healthcare and often located in rural areas are likely to be overcrowded and thus score higher on DEA scales. Technical efficiency scores in higher level facilities such as hospitals are likely to be significantly different from results of this study.

Findings of previous studies on technical efficiency in Ghana corroborate the results of this current study demonstrating the high levels of inefficiencies in healthcare facilities. Previous findings on Ghana showed that 35 % of 89 health facilities and 22 % of 113 health centres were optimally efficient [18, 19]. Other studies on technical efficiency in Ghana [4, 20] and some African countries [21–23, 25] found that less than 50 % of surveyed health facilities were efficient. While acknowledging the limitations associated with the DEA approach, conclusions in this paper are motivated by the widely recognized advantages of the DEA approach over other options [4, 20, 30].

Conclusions on technical efficiency levels in private and public health facilities vary in the literature depending on the methodology used and study setting. This study found that out of the 20 efficient facilities, 10 (50 %) were Public/government owned; 8 (40 %) were Private-for-profit, and 2 (10 %) were Mission/NGO facilities. In relative terms, this suggests lower levels of efficiency in mission/NGO and private-for-profit health facilities than public/government facilities. It was also found that higher efficiency scores were associated with public/government and Mission/NGO facilities in WR relative to those located in GAR (p < 0.05) (see Table 4). This suggests that the administrative region in which a private or public facility operates potentially associates with efficiency levels; however, concrete conclusions cannot be drawn in this paper because more detailed information on other vital performance indicators were not explored.

In terms facility ownership, the mean variable returns to scale (VRS) values showed that private-for-profit health facilities operating below the 1.0 (100 %) efficiency benchmark could improve their efficiency levels by increasing outputs to about 87 %. This observation is in contrast with findings of previous studies which indicated that private-for-profit health facilities are more efficient in health service delivery than public health facilities [33]. A more recent publication by Jehu-Appiah et al. [4] however confirm our findings. Jehu-Appiah et al. [4] compared technical efficiency levels in public/government, private-for-profit, mission and quasi-government facilities in Ghana and found that efficiency scores were relatively lower in private-for-profit facilities. Quasi-government facilities recorded higher efficiency scores followed by public/government and mission facilities.

A similar study by Akazili et al. [18] found that 65 % of 89 public health centres were inefficient relative to their peers, but no direct comparison was made with private-for-profit facilities. After conducting a meta-analysis of 317 publications on technical efficiency, Hollingsworth and Wildman [34] concluded that public facilities could potentially be more efficient in health service delivery than private-for-profit facilities.

Though the current study did not explore direct reasons for these differences in efficiency levels, a probable explanation would be that most private-for-private facilities are located in urban areas and better endowed with material and human resources which could result in redundancy and under-utilization of excess available resources. Over 60 % of NHIS-accredited private facilities are located in the two most urbanized cities in Ghana (Accra and Kumasi) [15]. This study observed that even though private facilities generally had more clinical staff, support staff, beds and wards than their public counterparts, output in terms of monthly SVDs, antenatal, postnatal, and reproductive health visits were lower, suggesting some level of redundancy in the service delivery system.

Given the increasing preferences for private health facilities over public due to perceived better quality in the former [35–37], the private sector has a competitive advantage over the public to maximize profit by instilling waste reduction strategies in the service delivery process. With the introduction of the NHIS, private-for-profit health facilities have opportunities to expand service coverage and improve efficiency levels because insured clients can access healthcare without considering the cost, a phenomenon that previously resulted in lower outpatient attendance in private-for-profit facilities [4].

Moreover, since input decreases by transfer of human and material resources from inefficient private-for-profit facilities to public/government facilities might not be a realistic intervention, output increases through outreach services and client-focused activities will be potentially more appropriate. Continuous quality care improvement by private-for-profit facilities will attract and retain clients and ultimately increase their customer base and reduce redundancies.

In addition, through effective public–private-partnership (PPP), the Ministry of Health (MoH), Ghana Health Service (GHS) and the NHIA could collaborate with private-for-profit facilities that are better endowed in material and human resources to render referral services to clients from public facilities. This form of agreement could help private facilities maximize available resources and reduce burden on public facilities. Some form of collaboration already exists where immunizations and other forms of child welfare services are done by GHS staff in private facilities on selected days. This partnership could be extended to include other service areas especially in communities where a private-for-profit facility is the sole source of healthcare. The MoH currently supports mission/NGO facilities by paying salaries of health staff on secondment by the GHS and this could be discussed for possible replication for private-for-profit health facilities. This will promote the financial viability of private facilities and motivate them to extend services to rural areas and expand their scope of healthcare delivery.

In the case of inefficient public/government facilities, since closure will not be a practical intervention, downsizing could be done by transferring excess staff to other public facilities with staff shortage. Likewise, clinic beds could be relocated or expansion projects diverted to facilities in greater need. These actions could help reduce inefficiency disparities and promote universal access to basic health services. However, these interventions should be preceded by comprehensive analysis of the quality care situation in these facilities in order not to compromise quality health service delivery emanating from inputs reductions.

This study found that technical efficiency did not significantly correlate with many quality care proxies per the NHIA and the *SafeCare Essentials* assessment tools. This suggests the need to exercise a fair balance between quality care and efficiency in health facilities since the optimal presence of one might not necessarily guarantee the existence of the other. Mainly improving efficiency levels without corresponding quality improvement plans could compromise quality care and render high efficiency gains meaningless. It is therefore important that these two components are equally emphasized.

The need to maintain a balance between quality and efficiency in NHIS-accredited health facilities is particularly vital because sacrificing one for the other could result in clients’ dissatisfaction with service quality leading to low confidence in the formal healthcare system. Results of the Spearman correlation test puts into perspective the technical efficiency performance of the sampled health facilities; the negative correlation between technical efficiency and overall NHIA assessment score (Coef. = −0.3158, p < 0.05) imply that quality care standards could have been compromised in health facilities deemed technically efficient because of limited material and human resources.

Even though there is sometimes a tradeoff between quality care and efficiency, the scope of this paper did not include a quality-adjusted DEA analysis. In the light of this, future research endeavors could incorporate quality care markers into DEA analysis to help policy makers identify and manage facilities that are: efficient with high quality; efficient with low quality; inefficient with high quality, and inefficient with low quality.

The policy recommendations proposed in this paper should be adequately juxtaposed with the quality standards in the pertinent health facilities. Health facilities deemed inefficient but provide better quality care might not necessarily have to reduce their input factors because that could compromise quality care standards. Instead, strategies to increase service output (i.e. outreach services, client-focused activities and health education) could be intensified to reduce redundancies.

Inefficient health facilities with low quality care standards might need more comprehensive interventions that involve internal reshuffling of redundant resources, increment in service output, staff capacity building on efficient utilization of resources, and effective implementation of quality improvement plans. Even though inefficient facilities theoretically have to reduce inputs and increase service output to attain optimal efficiency, if such facilities maintain acceptable quality care standards there might not be the need to reduce input factors since that could practically compromise quality care delivery.

Overall, the prominent message of this paper is that significant benefits, including cost savings, could be made by the NHIA and the MoH at large if accredited facilities operate more efficiently while maintaining acceptable quality care standards. The NHIA claims payment trend show that total claims payment rose from approximately US$ 8million in the year 2005 to over US$ 300 million in 2012 [5]. This unsustainable trend could be controlled if NHIS-accredited service providers operate more efficiently without compromising good quality care standards.

Key objectives of the capitation system being planned for nationwide roll-out by the NHIA include improvement in cost containment and sustainability of the NHIS through enhanced efficiency, quality care and more rational use of resources [5]. To accomplish these objectives and more, the NHIA and its stakeholders are encouraged to prioritize efficiency in quality care delivery on their policy agenda with particular attention to urban healthcare facilities where efficiency levels were relatively lower.

## Limitations

This study focused on only NHIS-accredited clinics and health centres thus possibly losing out valuable information on non-accredited health facilities. In addition, the study was conducted in two (2) out of ten (10) regions in Ghana engaging only 64 out of over 1000 accredited clinics and health centres nationwide. Extrapolation of the findings to other regions could therefore be a challenge. Finally, the study did not exhaust the entire concept of efficiency since only technical efficiency was assessed. Allocative efficiency was not measured because of limited data on revenue and cost of services in the 64 facilities.

## Conclusion

Out of the 64 sampled facilities, 20 (31 %) attained the 100 % efficiency benchmark; many Public/government facilities were found to be more efficient than private-for-profit and mission/NGO facilities. Even though this percentage might not necessarily reflect the absolute efficiency performance of health facilities in Ghana, the findings are relevant to inform policy on effective allocation, distribution and utilization of available health sector resources without compromising quality care standards. Ultimate policy decisions on inputs reductions and/or output increases in inefficient health facilities should be informed by reality checks on quality care standards in health facilities. This will avoid worsening quality care standards in the pursuit for high efficiency levels in health facilities.

High levels of inefficiencies and poor quality care standards in accredited health facilities could have dire consequences on the operational and financial sustainability of Ghana’s NHIS hence the need to prioritize technical efficiency as an NHIS sustainability strategy.

Perpetual inefficient management of limited resources coupled with poor quality care has the tendency to worsen existing challenges in attaining universal access to basic healthcare. Findings of this study are expected to kindle policy discourses on the need to complement quality improvement efforts with structured technical efficiency assessment in health facilities, including NHIS-accredited facilities.

### Policy implications/highlights

#### Commitment to equitable allocation and distribution of health resources

National policies aimed at bridging development gaps between rural and urban regions could help attract and retain qualified personnel in rural regions and reduce redundancies in urban-based health facilities which were found to be predominantly inefficient in this study. Comprehensive situational analysis of health needs in rural and urban areas will help attain equity in resource allocation.

#### Motivation for health workers in deprived areas

Another key intervention to improve efficiency will be re-designing of provider level incentives (not necessarily monetary) to attract and retain qualified health personnel in deprived regions where their services are most needed. Effective health worker incentives will not only contribute to quality improvement but also ensure efficient operation of health facilities. Effective incentive systems in the forms of staff accommodation, transportation and career development opportunities could help improve health worker density in less endowed regions and promote universal access to quality service delivery.

#### Improved client-centered healthcare system

Intensified public health education activities, client-centered strategies, quality care, and outreach campaigns could go a long way to increase output activities and reduce redundancies. Recent publications by the authors [22, 39] highlight the increasing relevance of client-centered quality care towards a sustainable health insurance system in Ghana and Africa at large.

#### Integration of efficiency assessment into mainstream monitoring and peer reviews

As part of the NHIA routine support to health facilities to improve performance, comprehensive technical efficiency assessment should be instituted in collaboration with the GHS, Society of Private Medical and Dental Practitioners (SPMDP), the Christian Health Association of Ghana (CHAG) and other religious bodies. This will help identify and assist inefficient health facilities re-allocate excess resources or increase service activities to instill efficiency in health service delivery. Likewise, best practices in efficient facilities could be learnt by their peers through joint peer-review activities.

#### Adopting technical efficiency as part of accreditation requirements

Future revision of the NHIA accreditation tools could incorporate mainstream technical efficiency variables to form part of the requirement for accreditation. This will encourage health facilities to strive for efficiency in their operations and still maintain acceptable quality care standards.

## Abbreviations

ANC: antenatal care
CHAG: Christian Health Association of Ghana
CHPS: Community-based health planning and services
CRS: Constant return to scale
DEA: data envelopment analysis
DEAP: data envelopment analysis programme
DHMTs: district health management teams
DMUs: Decision making units
ERC: Ethical review committee
FP: family planning
GAR: Greater Accra region
GHS: Ghana health service
MDGs: millennium development goals
MoH: Ministry of Health
NGO: non-governmental organization
NHIA: National Health Insurance Authority
NHIS: National Health Insurance Scheme
PCA: Principal Component Analysis
PNC: post–natal care
PPP: public private partnership
RCH: reproductive and child health
RCT: randomized control trial
RHA: regional health administration
SA^+^: Situational analysis plus
SPMDP: Society of private medical and dental practitioners
TE: technical efficiency
VRS: variable return to scale
WHO: World health organization
WR: Western region
